# Adult height and risk of breast cancer: a possible effect of early nutrition

**DOI:** 10.1054/bjoc.2001.1946

**Published:** 2001-09

**Authors:** T I Lund Nilsen, L J Vatten

**Affiliations:** Department of Community Medicine and General Practice, Norwegian University of Science and Technology, University Medical Centre, Trondheim, N-7489; Norwegian Cancer Society, PO Box 5327 Majorstua, Oslo, N-0304, Norway

**Keywords:** breast cancer, body height, nutrition, intrauterine exposures, Norway

## Abstract

The relationship of breast cancer to early reproductive development and height suggests that fetal and childhood nutrition may be important in its aetiology. Caloric restriction sufficient to reduce adult height may reduce breast cancer risk. During World War II (WWII) there was a marked reduction in average caloric intake in Norway that resulted in greater nutritional diversity. We hypothesized that a positive association between height and risk of breast cancer would be stronger among women who were born during this period than among women born before or after the war. A total of 25 204 Norwegian women were followed up for approximately 11 years, and 215 incident cases of breast cancer were registered. We found the strongest positive association between height and breast cancer among women born during WWII: women in the tallest tertile (>167 cm) had a relative risk of 2.5 (95% confidence interval = 1.2–5.5) compared with the shortest (≤ 162 cm). Among women born before or after the war we found no clear association with height. The association with height in the WWII cohort may imply a role for early nutrition in breast cancer aetiology. © 2001 Cancer Research Campaignhttp://www.bjcancer.com


*British Journal of Cancer* (2001) **85**(7), 959–961
© 2001 Cancer Research Campaign
doi: 10.1054/ bjoc.2001.1946, available online at http://www.idealibrary.com on IDEAL

### Adult height and risk of breast cancer: a possible effect of early nutrition

**TI Lund Nilsen[1,2] and LJ Vatten[1]**

[1]Department of Community Medicine and General Practice, Norwegian University of Science and Technology, University Medical Centre, N-7489 Trondheim; and [2]Norwegian Cancer Society, PO Box 5327 Majorstua, N-0304 Oslo, Norway

**Summary** The relationship of breast cancer to early reproductive development and height suggests that fetal and childhood nutrition may be important in its aetiology. Caloric restriction sufficient to reduce adult height may reduce breast cancer risk. During World War II (WWII) there was a marked reduction in average caloric intake in Norway that resulted in greater nutritional diversity. We hypothesized that a positive association between height and risk of breast cancer would be stronger among women who were born during this period than among women born before or after the war. A total of 25 204 Norwegian women were followed up for approximately 11 years, and 215 incident cases of breast cancer were registered. We found the strongest positive association between height and breast cancer among women born during WWII: women in the tallest tertile (>167 cm) had a relative risk of 2.5 (95% confidence interval = 1.2–5.5) compared with the shortest (≤ 162 cm). Among women born before or after the war we found no clear association with height. The association with height in the WWII cohort may imply a role for early nutrition in breast cancer aetiology. © 2001 Cancer Research Campaign http://www.bjcancer.com

**Keywords**: breast cancer; body height; nutrition; intrauterine exposures; Norway

Early menarche (Kelsey et al, 1993) and adult stature (de Waard, 1975; Tretli, 1989; Vatten and Kvinnsland, 1990) are positively associated with breast cancer risk. In populations characterized by nutritional diversity, differences in adult height may reflect differences in childhood nutrition (Willett, 1998). The relationship to early reproductive development and adult height may therefore indicate that nutrition at a young age can be important for future breast cancer risk (MacMahon, 1975).

During gestation, maternal nutritional deprivation may restrict fetal (Godfrey et al, 1996; Thame et al, 1997) and childhood growth (Cacciari et al, 2000), and the offspring may end up shorter as adults than their genetic potential would indicate (Karlberg and Luo, 2000; Luo et al, 2000; Rona and Chinn, 1995). Some studies have shown a positive association between birth weight and future risk of breast cancer (Ekbom et al, 1992; Michels et al, 1996; Sanderson et al, 1996; Stavola et al, 2000; Kaijser et al, 2001). Other studies have, however, shown no association between indicators of birth size and risk of breast cancer (Ekbom et al, 1997; Le Marchand et al, 1988).

In Norway, there was an overall reduction in average caloric intake during World War II, from 3475 kcal daily in 1939, to a minimum of 2700 kcal in late 1944 and early 1945 (Galtung-Hansen, 1947; Strøm, 1948). Among women who were born during that period, nutritional diversity was likely to be greater, both during intra-uterine life and early childhood, than among women who were born before or after the war. We therefore hypothesized that a positive association between height and risk of breast cancer would be stronger among women who were born during World War II. We used information from a cohort of 25 204 Norwegian women to test this hypothesis in a prospective study.

*Received 18 January 2001*
*Revised 5 June 2001*
*Accepted 5 June 2001*

*Correspondence to:* TI Lund Nilsen

### MATERIALS AND METHODS

This study includes 25 204 women born between 1925 and 1965 who participated in the Nord-Trøndelag Health Survey between 1984 and 1986 (Holmen and Midthjell, 1990). All residents in Nord-Trøndelag country aged 20 years or older were invited to participate in a brief health examination that included anthropometric measurements and self-administered questionnaires that provided information on medical history and lifestyle factors.

The participants were followed from the date of entry to the study (between January 1984 to April 1986) until the date of cancer diagnosis (of all sites), death, emigration, or the cut-off date of 1 January 1996, whichever occurred first. The unique identification number of every citizen in Norway enabled linkage to the national Cancer Registry in order to identify incident cases of cancer. To achieve a high degree of completeness and data quality, the material of the Cancer Registry is matched against the Register of Deaths at Statistics Norway. For all cancer cases registered since 1953, 84.7% are histologically verified and only 1.7% of the diagnoses are based on death certificate alone (Cancer Registry of Norway, 1998).

We used the Cox proportional hazards model (Kleinbaum, 1995) to compute incidence rate ratios (RRs) of breast cancer associated with adult height by year of birth. Height was categorized into tertiles according to the distribution of the total population (cut-off = 162 cm and 167 cm), while year of birth was categorized into five birth cohorts. The first birth cohort ranged from January 1925 to December 1929, and the second from January 1930 to December 1934. Since Norway was occupied between 9 April 1940 and 8 May 1945, the fourth birth cohort (i.e. the World War II cohort) ranged from July 1940 to December 1945, and consequently the third ranged from January 1935 to June 1940. The last birth cohort included all women born in January 1946 or later. In the stratified analysis of these youngest women (born in 1946 or later) we adjusted for age at study entry in three categories (< 30, 30–34, and ≥ 35 years). Further multivariate analyses were conducted to assess potential confounding by body mass index (BMI), smoking, and physical activity.

All statistical analyses were performed using the statistical software SPSS for Windows (Release 10.0.5, Copyright © SPSS Inc., 1989–1999).

### RESULTS

During almost 12 years of follow-up (median = 11.0 years) we observed a total of 276 239 person-years, and 215 incident cases of breast cancer were registered. Mean age at study entry was 39.5 years (range = 20–61 years) and mean age at diagnosis of breast cancer was 53.4 years (range = 31–70 years).

We found no independent effect of height on the risk of breast cancer in the total population (age-adjusted *P*-trend = 0.6). However, in analyses stratified by birth cohort we found a strong positive association with height among the 3792 women (43 cases) who where born during World War II, where women in the tallest tertile of height (> 167 cm) had more than twice the risk of breast cancer compared with women in the shortest tertile (≤ 162 cm) (RR = 2.5; 95% CI = 1.2–5.5) (Table 1). For women in the other birth cohorts there was no significant association between height and breast cancer risk. Adjustment for BMI, smoking and physical activity did not change these results (data not shown).

### DISCUSSION

In this prospective study of Norwegian women, we found that an association between adult height and breast cancer risk was confined to women who were born during World War II. Women in the highest tertile of height had more than twice the risk of breast cancer compared with women in the lowest tertile. Among women born before or after the war we found no clear association with height.

We adjusted for differences in body mass index, smoking, and physical activity. However, we had no information on age at menarche, age at first full-term pregnancy, and parity, and this may be a weakness of our study. Also, by stratifying cases according to birth cohorts, the number of cases within each category of height was small, thereby reducing the precision of the results.

Recently, the association between indicators of birth size and breast cancer risk has received attention, and some studies have reported a positive association with birth weight (Ekbom et al, 1992; Michels et al, 1996; Sanderson et al, 1996; Stavola et al, 2000; Kaijser et al, 2001). Others, however, have not been able to confirm this finding (Ekbom et al, 1997; Le Marchand et al, 1988). For birth length or placenta weight, there have been no clear findings, but pre-eclampsia in the mother, which is associated with reduced fetal growth, appears to reduce the risk of breast cancer in the daughters (Ekbom et al, 1992; Ekbom et al, 1997; Sanderson et al, 1998).

In a previous Norwegian cohort study, there was a positive association between height and breast cancer risk among women whose peripubertal growth coincided with World War II (Vatten and Kvinnsland, 1990). Also, a large ecological study in Norway found that the incidence of breast cancer was lower than expected among women who experienced puberty during the war (Tretli and Gaard, 1996). In the present cohort we found no significant association with height among women who were born in the late 1920s or early 1930s, as would be predicted from the other two Norwegian studies. The cancer cases contributing to the effect of WWII in the present study and in the study by Vatten and Kvinnsland were mainly pre-menopausal. Tretli (1989) found, however, that the association between height and breast cancer did not substantially differ by age at diagnosis.

If fetal or childhood growth is important for a girl's prospects of developing breast cancer, one important set of biological mediators may be the insulin-like growth factors (IGFs) (Smith et al, 2000). The IGFs play an important role during the peripubertal growth spurt, and tallness is positively associated with plasma IGF-I levels (Juul et al, 1994). It is, however, unclear whether differences in IGF levels during early development are reflected in differences in adult IGF. Recently, two prospective studies of IGF-I measured in adult women found a positive association with risk of premenopausal, but not postmenopausal breast cancer (Hankinson et al, 1998; Toniolo et al, 2000).

In summary, this study shows a strong positive association between adult height and risk of breast cancer among the women

**Table 1** Rate ratio (RR) with 95% confidence interval (CI) of breast cancer risk associated with tertiles of height stratified by birth cohort

| Birth cohort | Tertiles of height[a] | No. of person-years | No. of cases | RR | 95% CI |
|---|---|---|---|---|---|
| 1925–1929 | T1 | 16 793 | 37 | 1.0 | – |
| | T2 | 9 467 | 20 | 0.9 | 0.5–1.6 |
| | T3 | 4 870 | 6 | 0.5 | 0.2–1.1 |
| 1930–1934 | T1 | 13 783 | 18 | 1.0 | – |
| | T2 | 9 282 | 12 | 0.9 | 0.5–2.0 |
| | T3 | 5 454 | 7 | 0.9 | 0.4–2.3 |
| 1935–1940[b] | T1 | 11 933 | 10 | 1.0 | – |
| | T2 | 11 510 | 8 | 0.8 | 0.3–1.9 |
| | T3 | 8 416 | 10 | 1.3 | 0.5–3.1 |
| 1940–1945[b] | T1 | 13 709 | 9 | 1.0 | – |
| | T2 | 15 189 | 12 | 1.1 | 0.5–2.7 |
| | T3 | 12 770 | 22 | 2.5 | 1.2–5.5 |
| ≥ 1946[c] | T1 | 38 439 | 11 | 1.0 | – |
| | T2 | 47 629 | 16 | 1.1 | 0.5–2.4 |
| | T3 | 52 165 | 16 | 1.0 | 0.5–2.3 |

[a]Cut-off for tertiles of height = 162 cm and 167 cm; [b]Cfr. time cut-off in 1940; see Materials and methods; [c]RR is adjusted for age at study entry (< 30, 30–34, and ≥ 35 years).

*© 2001 Cancer Research Campaign*

whose fetal growth coincided with the years of World War II. This period was characterized by general caloric restriction (Galtung-Hansen, 1947; Strøm, 1948) and a deceleration in the secular height gain observed before the war (Brundtland et al, 1980). We suggest that the strong association with height may reflect greater nutritional diversity during gestation among the mothers, implying a role for early nutrition in the aetiology of breast cancer.

### ACKNOWLEDGEMENTS

This research is based on data made available by the National Health Screening Service, The Cancer Registry of Norway, and the National Institute of Public Health, Community Medicine Research Centre in Verdal, Nord-Trøndelag County, Norway.

TI Lund Nilsen is a recipient of a research fellowship from the Norwegian Cancer Society, Oslo, Norway.

### REFERENCES

Brundtland GH, Liestol K and Walloe L (1980) Height, weight and menarcheal age of Oslo schoolchildren during the last 60 years. *Ann Hum Biol* **7**: 307–322

Cacciari E, Salardi S, David C, Tassinari D, Dalla CC, Pilu GL, Mainetti B, Gualandi S and Bovicelli L (2000) Is statural growth predictable in utero? Follow-up from the second trimester of gestation to the 8th year of life. *J Pediatr Endocrinol Metab* **13**: 381–386

Cancer Registry of Norway (1998) *Cancer in Norway 1995*. Cancer Registry of Norway: Oslo de Waard F (1975) Breast cancer incidence and nutritional status with particular – to body weight and height. *Cancer Res* **35**: 3351–3356

Ekbom A, Hsieh CC, Lipworth L, Adami HQ and Trichopoulos D (1997) Intrauterine environment and breast cancer risk in women: a population-based study. *J Natl Cancer Inst* **89**: 71–76

Ekbom A, Trichopoulos D, Adami HO, Hsieh CC and Lan SJ (1992) Evidence of prenatal influences on breast cancer risk. *Lancet* **340**: 1015–1018

Galtung-Hansen O (1947) Food conditions in Norway during the war 1939–1945. *Proc Nutr Soc* **5**: 263

Godfrey K, Robinson S, Barker DJ, Osmond C and Cox V (1996) Maternal nutrition in early and late pregnancy in relation to placental and fetal growth. *BMJ* **312**: 410–414

Hankinson SE, Willett WC, Colditz GA, Hunter DJ, Michaud DS, Deroo B, Rosner B, Speizer FE and Pollak M (1998) Circulating concentrations of insulin-like growth factor-I and risk of breast cancer. *Lancet* **351**: 1393–1396

Holmen J and Midthjell K (1990) *The North-Trøndelag health survey 1984–86: purpose, background and methods: participation, non-participation and frequency distributions*. Statens institutt for folkehelse: Oslo

Juul A, Bang P, Hertel NT, Main K, Dalgaard P, Jorgensen K, Muller J, Hall K and Skakkebaek NE (1994) Serum insulin-like growth factor-I in 1030 healthy children, adolescents, and adults: relation to age, sex, stage of puberty, testicular size, and body mass index. *J Clin Endocrinol Metab* **78**: 744–752

Kaijser M, Lichtenstein P, Granath F, Erlandsson G, Cnattingius S and Ekbom A (2001) In utero exposures and breast cancer: a study of opposite-sexed twins. *J Natl Cancer Inst* **93**: 60–62

Karlberg J and Luo ZC (2000) Foetal size to final height. *Acta Paediatr* **89**: 632–636

Kelsey JL, Gammon MD and John EM (1993) Reproductive factors and breast cancer. *Epidemiol Rev* **15**: 36–47

Kleinbaum DG (1995) *Survival analysis: a self-learning text*. Springer-Verlag: New York

Le Marchand L, Kolonel LN, Myers BC and Mi MP (1988) Birth characteristics of premenopausal women with breast cancer. *Br J Cancer* **57**: 437–439

Luo ZC, Low LC and Karlberg J (2000) Fetal size to final height in Hong Kong Chinese children. *J Pediatr Endocrinol Metab* **13**: 269–279

MacMahon B (1975) Formal discussion of breast cancer incidence and nutritional status with particular – to body weight and height. *Cancer Res* **35**: 3357

Michels KB, Trichopoulos D, Robins JM, Rosner BA, Manson JE, Hunter DJ, Colditz GA, Hankinson SE, Speizer FE and Willett WC (1996) Birthweight as a risk factor for breast cancer. *Lancet* **348**: 1542–1546

Rona RJ and Chinn S (1995) Genetic and environmental influences on growth. *J Med Screen* **2**: 133–139

Sanderson M, Williams MA, Daling JR, Holt VL, Malone KE, Self SG and Moore DE (1998) Maternal factors and breast cancer risk among young women. *Paediatr Perinat Epidemiol* **12**: 397–407

Sanderson M, Williams MA, Malone KE, Stanford JL, Emanuel I, White E and Daling JR (1996) Perinatal factors and risk of breast cancer. *Epidemiology* **7**: 34–37

Smith GD, Gunnell D and Holly J (2000) Cancer and insulin- like growth factor-I. A potential mechanism linking the environment with cancer risk. *BMJ* **321**: 847–848

Stavola BL, Hardy R, Kuh D, Silva IS, Wadsworth M and Swerdlow AJ (2000) Birthweight, childhood growth and risk of breast cancer in a British cohort. *Br J Cancer* **83**: 964–968

Strøm A (1948) Examination into the diet of Norwegian families during the war-years 1942–1945. *Acta Med Scand* **214** (Suppl)

Thame M, Wilks RJ, McFarlane-Anderson N, Bennett FI and Forrester TE (1997) Relationship between maternal nutritional status and infant's weight and body proportions at birth. *Eur J Clin Nutr* **51**: 134–138

Toniolo P, Bruning PF, Akhmedkhanov A, Bonfrer JM, Koenig KL, Lukanova A, Shore RE and Zeleniuch-Jacquotte A (2000) Serum insulin-like growth factor-I and breast cancer. *Int J Cancer* **88**: 828–832

Tretli S (1989) Height and weight in relation to breast cancer morbidity and mortality. A prospective study of 570,000 women in Norway. *Int J Cancer* **44**: 23–30

Tretli S and Gaard M (1996) Lifestyle changes during adolescence and risk of breast cancer: an ecologic study of the effect of World War II in Norway. *Cancer Causes Control* **7**: 507–512

Vatten LJ and Kvinnsland S (1990) Body height and risk of breast cancer. A prospective study of 23,831 Norwegian women. *Br J Cancer* **61**: 881–885

Willett W (1998) *Nutritional epidemiology*. Oxford University Press: New York

© 2001 Cancer Research Campaign
